# Health literacy and public health: A systematic review and integration of definitions and models

**DOI:** 10.1186/1471-2458-12-80

**Published:** 2012-01-25

**Authors:** Kristine Sørensen, Stephan Van den Broucke, James Fullam, Gerardine Doyle, Jürgen Pelikan, Zofia Slonska, Helmut Brand

**Affiliations:** 1 Department of International Health, Research School of Primary Care and Public Health, Maastricht University, Maastricht, The Netherlands; 2 Université Catholique de Louvain, Louvain-la-Neuve, Belgium; 3 University College Dublin, National University of Ireland, Dublin, Ireland; 4 Ludwig Boltzmann Institute Health Promotion Research, Vienna, Austria; 5 National Institute of Cardiology, Warsaw, Poland; 6 The HLS-EU Consortium, Maastricht University, Maastricht, the Netherlands

## Abstract

**Background:**

Health literacy concerns the knowledge and competences of persons to meet the complex demands of health in modern society. Although its importance is increasingly recognised, there is no consensus about the definition of health literacy or about its conceptual dimensions, which limits the possibilities for measurement and comparison. The aim of the study is to review definitions and models on health literacy to develop an integrated definition and conceptual model capturing the most comprehensive evidence-based dimensions of health literacy.

**Methods:**

A systematic literature review was performed to identify definitions and conceptual frameworks of health literacy. A content analysis of the definitions and conceptual frameworks was carried out to identify the central dimensions of health literacy and develop an integrated model.

**Results:**

The review resulted in 17 definitions of health literacy and 12 conceptual models. Based on the content analysis, an integrative conceptual model was developed containing 12 dimensions referring to the knowledge, motivation and competencies of accessing, understanding, appraising and applying health-related information within the healthcare, disease prevention and health promotion setting, respectively.

**Conclusions:**

Based upon this review, a model is proposed integrating medical and public health views of health literacy. The model can serve as a basis for developing health literacy enhancing interventions and provide a conceptual basis for the development and validation of measurement tools, capturing the different dimensions of health literacy within the healthcare, disease prevention and health promotion settings.

## Background

Health literacy is a term introduced in the 1970s [1] and of increasing importance in public health and healthcare. It is concerned with the capacities of people to meet the complex demands of health in a modern society [2]. Health literate means placing one's own health and that of one's family and community into context, understanding which factors are influencing it, and knowing how to address them. An individual with an adequate level of health literacy has the ability to take responsibility for one's own health as well as one's family health and community health [3].

It is important to distinguish health literacy from literacy in general. According to the United Nation Education, Science and Culture Organization (UNESCO) during its history in English, the word 'literate' mostly meant to be 'familiar with literature' or in general terms 'well educated, learned'. While maintaining its broader meaning of being knowledgeable or educated in a particular area, during the late nineteenth century it has also come to refer to the abilities to read and write text. In recent years four understandings of literacy have appeared from the debate of the notion: 1) Literacy as an autonomous set of skills; 2) literacy as applied, practiced and situated; 3) literacy as a learning process; and 4) literacy as text. The focus is furthermore broadening so that literacy is not only referring to individual transformation, but also to contextual and societal transformation in terms of linking health literacy to economic growth and socio-cultural and political change [4].

The same development can be traced in the realm of health literacy. For some time most emphasis was given to health literacy as the ability to handle words and numbers in a medical context, and in recent years the concept is broadening to also understanding health literacy as involving the simultaneous use of a more complex and interconnected set of abilities, such as reading and acting upon written health information, communicating needs to health professionals, and understanding health instructions [5]. American studies in the 1990s linked literacy to health, showing an association between low literacy and decreased medication adherence, knowledge of disease and self-care management skills [6]. The 2003 National Assessment of Adult Literacy (NAAL), which measured the English literacy of American adults (people age 16 and older) included questions related to health, and revealed the consequences of limited literacy on health and healthcare [7].

A report from the Institute of Medicine indicates that nearly half of the American adult population may have difficulties in acting on health information [8]. This finding has been referred to as the "health literacy epidemic" [9]. In response, measures have been taken to ensure better health communication through establishing health literacy guidelines [10], and a trans-disciplinary approach has been encouraged to improve health literacy [11]. To support this approach, the American Medical Association recommends four areas for research: health literacy screening; improving communication with low-literacy patients; costs and outcomes of poor health literacy; and causal pathways of how poor health literacy influences health [12,13]. The research literature on health literacy has expanded exponentially, with nearly 5,000 PubMed-listed publications to date (Primo November 2011), the majority of which have been published since 2005 [5,14] and is evident that health literacy is being explored within different disciplines and with different approaches, e.g. looking at the role of health educators in promoting health literacy [15]; public health literacy for lawyers [16], health communication [17], the prevalence of limited health literacy [18], and health literacy as an empowerment tool for low-income mothers [19].

While until recently the interest in health literacy was mainly concentrated in the United States and Canada, it has become more internationalized over the past decade [20]. Research on health literacy has taken place in e.g. Australia [21,22], Korea [23], Japan [24], the UK [25], the Netherlands [26], and Switzerland [27]. Although the EU produced less than a third of the global research on health literacy between 1991 and 2005 [28,29], the importance of the issue is increasingly recognized in European health policies. As a case in point, health literacy is explicitly mentioned as an area of priority action in the European Commission's Health Strategy 2008-2013 [30]. It is linked to the core value of citizen empowerment, and the priority actions proposed by the European Commission include the promotion of health literacy programs for different age groups.

However, with the proliferation of health literacy research and policy measures, it becomes clear that there is no unanimously accepted definition of the concept. Moreover, the constituent dimensions of health literacy remain disputed, and attempts to operationalize the concept vary widely in scope, method and quality. As a result, it is very difficult to compare findings with regard to health literacy emerging from research in different countries.

The current article aims to address this issue by offering a systematic review of existing definitions and concepts of health literacy as reported in the international literature, by identifying the central health literacy dimensions, the target group as well as antecedents and consequences if explained. in order to develop an integrated definition and conceptual model capturing the most comprehensive evidence-based dimensions of health literacy.

## Method

A systematic review in Medline, Pubmed and Web of Science was performed by two independent research teams in autumn 2009 and spring 2010 and the results compared and combined to obtain information regarding two research questions: (1) How is health literacy defined? and (2) How can health literacy be conceptualized? To retrieve studies, 17 keywords (definition, model, concept, dimension, framework, conceptual framework, theory, analysis, qualitative, quantitative, competence, skill, "public health", communication, information, functional, critical) were combined (using the Boolean operator *and*) with the search terms "health literacy", "health competence", and health competence (without quotes). Combinations of the keywords with health literacy (without quotes) produced a list of studies that was too wide for the purpose of this study, and therefore not used for the review. From the resulting list, studies were selected for inclusion in the review on the basis of their abstracts. Eligible studies were included which met the following inclusion criteria: (1) written in English; (2) concerned with health literacy in a developed country; and (3) offering relevant content with regard to the definition or conceptualization of health literacy, or a combination of these issues.

The eligible literature was scanned for definitions, and a content analysis was performed in three steps: Firstly, the definitions were coded and condensed by two research teams working independently. Secondly the analysis was discussed with a panel of health experts from the European Health Literacy Consortium. In a third step, the feedback was elaborated by the original research team and integrated in a final analysis yielding a condensed 'all-inclusive' definition of health literacy capturing the different meanings and dimensions presented in the literature. In addition, an overview of all models from the eligible literature was conducted, the models were compared according to dimensions, target groups and antecedents as well as consequences if explained, and as a result a new conceptual model was drafted capturing the most comprehensive core dimensions of health literacy identified as well as its antecedents and consequences.

## Results

The combination of the key words with the three search terms resulted in the initial identification of 170 publications. Additional publications were found by reference tracking and included in the review. Based on the application of the inclusion criteria to the abstracts, 19 publications were retrieved which explicitly dealt with the definition of health literacy, and 12 with conceptual frameworks of health literacy.

### Definitions of health literacy

From the 19 publications focusing specifically on definitions of health literacy 17 explicit definitions could be derived (Table 1). Of these definitions, the ones by the American Medical Association [12], the Institute of Medicine [8] and WHO [31] are cited most frequently in the eligible literature. A shared characteristic of these definitions is their focus on individual skills to obtain, process and understand health information and services necessary to make appropriate health decisions. However, recent discussions on the role of health literacy highlight the importance of moving beyond an individual focus, and of considering health literacy as an interaction between the demands of health systems and the skills of individuals. In fact the Institute of Medicine report already alluded that "health literacy is a shared function of social and individual factors, which emerges from the interaction of the skills of individuals and the demands of social systems" [8]. More recently, Kwan [32] and Pleasant [33] underscored the importance of skills and abilities on the part of *all *parties involved in communication and decisions about health, including patients, providers, health educators, and lay people. This broader view is presented in the definition proposed by Zarcadoolas, Pleasant and Greer [34], who state that a health literate person is able to apply health concepts and information to novel situations, and to participate in ongoing public and private dialogues about health, medicine, scientific knowledge, and cultural beliefs. Freedman and her collegues [35] argue that the medical perspective on factors influencing people's health should be shifted towards a societal level, and that a distinction must be made between public and individual health literacy. Public health literacy can be found when the conceptual foundations of health literacy are in place in a group or community.

**Table 1:** Definitions of health literacy

1	WHO (1998)	"The cognitive and social skills which determine the motivation and ability of individuals to gain access to understand and use information in ways which promote and maintain good health" [31]
2	American Medical Association's (1999)	"The constellation of skills, including the ability to perform basic reading and numeral tasks required to function in the healthcare environment" [12]

3	Nutbeam (2000)	"The personal, cognitive and social skills which determine the ability of individuals to gain access to, understand, and use information to promote and maintain good health" [36]

4	Institute of Medicine (2004)	"The individuals' capacity to obtain, process and understand basic health information and services needed to make appropriate health decisions" [8]

5	Kickbusch, Wait & Maag (2005)	"The ability to make sound health decision(s) in the context of everyday life--at home, in the community, at the workplace, the healthcare system, the market place and the political arena. It is a critical empowerment strategy to increase people's control over their health, their ability to seek out information and their ability to take responsibility" [37]

6	Zarcadoolas, Pleasant & Greer (2003, 2005, 2006)	"The wide range of skills, and competencies that people develop to seek out, comprehend, evaluate and use health information and concepts to make informed choices, reduce health risks ad increase quality of life" [34,38,39]

7	Paasche-Orlow & Wolf (2006)	"An individual's possession of requisite skills for making health-related decisions, which means that health literacy must always be examined in the context of the specific tasks that need to be accomplished. The importance of a contextual appreciation of health literacy must be underscored" [40]

8	EU (2007)	"The ability to read, filter and understand health information in order to form sound judgments" [30]

9	Pavlekovic (2008)	"The capacity to obtain, interpret and understand basic health information and services and the competence to use such information to enhance health" [41]

10	Rootman & Gordon-Elbihbety (2008)	"The ability to access, understand, evaluate and communicate information as a way to promote, maintain and improve health in a variety of settings across the life course" [42]

11	Ishikawa & Yano (2008)	"The knowledge, skills and abilities that pertain to interactions with the healthcare system" [14]

12	Mancuso (2008)	"A process that evolves over one's lifetime and encompasses the attributes of capacity, comprehension, and communication. The attributes of health literacy are integrated within and preceded by the skills, strategies, and abilities embedded within the competencies needed to attain health literacy" [43]

13	Australian Bureau of Statistics (2008)	"The knowledge and skills required to understand and use information relating to health issues such as drugs and alcohol, disease prevention and treatment, safety and accident prevention, first aid, emergencies, and staying healthy" [44]

14	Yost et al. (2009)	"The degree to which individuals have the capacity to read and comprehend health-related print material, identify and interpret information presented in graphical format (charts, graphs and tables), and perform arithmetic operations in order to make appropriate health and care decisions" [45]

15	Adams et al. (2009)	"The ability to understand and interpret the meaning of health information in written, spoken or digital form and how this motivates people to embrace or disregard actions relating to health" [22]

16	Adkins et al. (2009)	"The ability to derive meaning from different forms of communication by using a variety of skills to accomplish health-related objectives" [46]

17	Freedman et al. (2009)	"The degree to which individuals and groups can obtain process, understand, evaluate, and act upon information needed to make public health decisions that benefit the community" [35]

The content analysis on the definitions yielded six clusters representing: (1) competence, skills, abilities; (2) actions; (3) information and resources; (4) objective; (5) context; and (6) time as outlined in Table 2. Accordingly each cluster was carefully examined, discussed and condensed by the research team and the resulting chosen terms and notions were combined to yield a new 'all inclusive' comprehensive definition capturing the essence of the 17 definitions identified in the literature:

**Table 2:** The six clusters identified when condensing the definitions from the literature review

Competence / skills / abilities	Action	Information	Objective	Context	Time
Skills Possession of requisite skills/Constellation of skills/Wide range of skills Cognitive skills Social skills Personal skills The ability The capacity The knowledge The competencies Motivation Comprehension Communication	To gain access To understand To use To perform basic reading and numerical tasks To obtain To process To seek out To comprehend To evaluate To read To filter To find To appraise To communicate To interpret To identify To perform arithmetic operations To embrace or disregard actions To derive meaning To act To make sound decisions/to make health-related decisions To take responsibility To pertain interactions To attain capacity, comprehension and communication	Information Health information Information relating to health Basic health information Health-related print-material Information presented in graphical form Health information in written, spoken or digital form Different forms of communication Concepts Services	Promote and maintain good health To function in the health care environment To make appropriate health decisions A critical empowerment strategy to increase people's control over their health To make informed choices Reduce health risks Increase quality of life To form sound judgments To engage in demands of different health contexts To promote health To enhance health To improve health To make appropriate health and care decisions To accomplish health-related objectives To make public health decisions that benefit the community	Variety of settings The health care environment Different health contexts Health care setting Health related contexts The everyday life at home, in the community, at the workplace, within the healthcare system, at the market place and within the political arena HL always related to the context of the specific tasks needed to be accomplished	Across the life course Evolves over lifetime

*Health literacy is linked to literacy and entails people's knowledge, motivation and competences to access, understand, appraise, and apply health information in order to make judgments and take decisions in everyday life concerning healthcare, disease prevention and health promotion to maintain or improve quality of life during the life course*.

This definition encompasses the public health perspective and can easily be specified to accommodate an individual approach by substituting the three domains of health "healthcare, disease prevention and health promotion" with "being ill, being at risk and staying healthy".

### Concepts of health literacy

Table 3 lists the publications which provide a conceptual model of health literacy. From this overview, two issues become apparent. Firstly, health literacy is a multidimensional concept and consists of different components. Secondly, most conceptual models not only consider the key components of health literacy, but also identify the individual and system-level factors that influence a person's level of health literacy, as well as the pathways that link health literacy to health outcomes.

**Table 3:** Conceptual models of health literacy

	Reference	Dimensions	Antecedents	Consequences
1	Nutbeam (2000) [36]	- Functional health literacy - Interactive health literacy - Critical health literacy	Health promotion actions (education, social mobilization, advocacy)	*Individual benefits* - Improved knowledge of risks - Compliance with prescribed actions. Improved capacity to act independently on knowledge - Improved motivation and self-confidence - Improved individual resilience to adversity *Community/social benefits* - Increased participation in population health programs - Improved capacity to influence social norms and interact with social groups. - Improved capacity to act on social and economic determinants of health - improved community empowerment

2	Lee et al. (2004) [47]	- Disease and self-care knowledge. - Health risk behavior - Preventive care and physician visits. - Compliance with medications.	- Social-economic status - Gender - Ethnicity - Health insurance coverage - Disease severity - Income discrepancy - Ethnic composition of the community	- Health status - Emergency care - Hospitalization

3	Institute of Medicine (2004) [8]	- Cultural and conceptual knowledge - Listening - Speaking - Arithmetical skills - Writing skills - Reading skills	- Education, culture and language. - Communication and assessment skills of people with whom individuals interact for health - Ability of the media, the marketplace, and governmental agencies to provide health information in an appropriate manner	Health outcomes and costs

4	Zarcadoolas et al. (2005) [38]	- Fundamental literacy Science literacy - Civic literacy Cultural literacy	- Health status - Demographic, sociopolitical, psychosocial and cultural factors	- Ability to apply information to novel situations Ability to participate in public and private dialogues about health, medicine, scientific knowledge and cultural beliefs

5	Speros (2005) [48]	- Reading/numeracy skills - Comprehension - Capacity to use health information in decision making - Successful functioning in healthcare consumer role	- Literacy - Health-related experience.	- Improved self-reported health status - Lower healthcare costs - Increased health knowledge - Shorter hospitalization Less frequent use of healthcare services

6	Baker (2006) [49]	- Health-related print literacy - Health-related oral literacy.	- Health-related reading fluency - Health-related vocabulary - Familiarity with health concepts Complexity and difficulty of the printed and spoken messages in the healthcare environment	- Acquisition of new knowledge - More positive attitudes - Greater self-efficacy Positive health behaviors - Better health outcomes

7	Paashe-Orlow & Wolf (2007) [40]	- Listening - Verbal fluency - Memory span - Navigation.	- Socioeconomic status Occupation - Employment status Income - Social support - Culture and language - Education - Age - Race/ethnicity Personal competences such as vision, hearing, verbal ability, memory and reasoning.	- Access and utilization of healthcare (influenced by patients' navigation skills, self-efficacy and perceived barriers, and by system's complexity, acute care orientation and tiered delivery model). - Patient/provider interactions (influenced patients' knowledge, beliefs and participation in decision-making, and by providers' communication skills, teaching ability, time and patient-centered care). Self care (influenced by patients' motivation, problem-solving, self-efficacy, knowledge/skills, and by support technologies, mass media, health education and resources)

8	Kickbusch & Maag (2008) [2]	- Functional - Interactive - Critical	- Education system - Health-care system - Culture/home and community - Work - Politics Market	- Health outcomes and costs

9	Mancuso (2008) [43]	- Capacity - Comprehension Communication	- Operational competence - Interactive competence - Autonomous competence - Informational competence - Contextual competence - Cultural competence	- Healthcare costs - Knowledge of diseases and treatments - Self-management skills - Ability to care for chronic conditions - Compliance - Medical or medication treatment errors - Access to and use of healthcare services. - Use of expensive services such as emergency care and inpatient admissions. Prevention and screening health-promoting behaviors Health status, defined as physical illness or perceptions of illness, disease or impairment

10	Manganello (2008) [50]	- Functional health literacy - Interactive health literacy - Critical health literacy Media literacy	- Individual traits (age, race, gender, cultural background, cognitive and physical abilities, social skills) - Media use - Peer and parent influences - Mass media, the education system and the health system	- Health behavior - Health costs - Health service use

11	Freedman et al. (2009) [35]	- Conceptual foundations - Critical skills Civic orientation	Social, environmental and political forces	- Resolve some of society's more pressing health issues - Alleviate social injustices.

12	Von Wagner et al. (2009) [51]	- Ability to rely on literacy and numeracy skills when they are required to solve problems	- Epidemiological or structural determinants - Individual influences - Reading and arithmetic skills - External influences	- Access and use of healthcare - Patient-provider interaction - Management of health and illness

#### Dimensions of health literacy

The distinction between medical and public health literacy [35] is reflected in the identification of different dimensions. Within the definition of health literacy as individual capacities, the Institute of Medicine [8] consider cultural and conceptual knowledge, listening, speaking, arithmetical, writing, and reading skills as the main components of health literacy. Speros [48] also identifies reading and numeracy skills as the defining attributes, but adds comprehension, the capacity to use health information in decision making, and successful functioning in the role of healthcare consumer as dimensions. Baker [49] divides health literacy into health related print literacy and health related oral literacy, while Paashe-Orlow and Wolf [40] distinguish between listening, verbal fluency, memory span and navigation. Lee et al. [47] identify four interrelated factors: (1) disease and self-care knowledge; (2) health risk behavior; (3) preventive care and physician visits; and (4) compliance with medication. While these defining elements of health literacy vary considerably they all concern cognitive capabilities, skills and behaviors which reflect an individual's capacity to function in the role of a patient within the healthcare system.

Proponents of the population health literacy view, on the other hand, extend the concept to include dimensions which go beyond individual competences and the medical context. The prototypical model is that of Nutbeam [36], which distinguishes between three typologies of health literacy: (1) *Functional health literacy *refers to the basic skills in reading and writing that are necessary to function effectively in everyday situations, broadly comparable with the content of "medical" health literacy referred to above; (2) *Interactive health literacy *refers to more advanced cognitive and literacy skills which, together with social skills, can be used to actively participate in everyday situations, extract information and derive meaning from different forms of communication, and apply this to changing circumstance; and (3) *Critical health literacy *refers to more advanced cognitive skills which, together with social skills, can be applied to critically analyze information and use this to exert greater control over life events and situations. The different typologies represent levels of knowledge and skills that progressively support greater autonomy and personal empowerment in health related decision-making, as well as engagement with a wider range of health knowledge that extends from personal health management to the social determinants of health [52]. Manganello [50] adds media literacy as the ability to critically evaluate media messages. Zarcadoolas et al. [38] distinguish between *fundamental literacy *(skills and strategies involved in reading, speaking, writing and interpreting numbers); *science literacy *(the levels of competence with science and technology); *civic literacy *(abilities that enable citizens to become aware of public issues and become involved in the decision-making process); and *cultural literacy *(the ability to recognize and use collective beliefs, customs, world-view and social identity in order to interpret and act on health information). In a similar vein, Freedman et al. [35] identify three dimensions of public health literacy, each of which involves corresponding competences: (1) *Conceptual foundations *includes the basic knowledge and information needed to understand and take action on public health concerns; individuals and groups should be able to discuss core public health concepts, public health constructs and ecologic perspectives. (2) *Critical skills *relates to the skills necessary to obtain, process, evaluate, and act upon information that is needed to make public health decisions that benefit the community; an individual or group should be able to obtain, evaluate, and utilize public health information, identify public health aspects of personal and community concerns, and access who is naming and framing public health problems and solutions. (3) *Civic orientation *includes the skills and resources necessary to address health concerns through civic engagement; an individual or group should be able to articulate the uneven distribution of burdens and benefits of the society, evaluate who benefits and who is harmed by public health efforts, communicate current public health problems, and address public health problems through civic action, leadership, and dialogue. Mancuso [43] emphasizes that health literacy is a process that evolves over a person's lifetime and identify the attributes of health literacy to be capacity, comprehension and communication. (1) The *Capacity skills *related to health literacy include gathering, analyzing, and evaluating health information for credibility and quality, working together, managing resources, seeking guidance and support, developing and expressing a sense of self, creating and pursuing a vision and goals, and keeping pace with change. Oral language skills are also considered essential. Social skills and credentials such as reading, listening, analytical, decision-making, and numerical abilities are important as well to advocate for oneself, to act on health information, and to negotiate and navigate within the health-care system. (2) *Comprehension *is a complex process based on the effective interaction of logic, language, and experience and is crucial to the accurate interpretation of a myriad of information that is provided to the modern patient, such as discharge instructions, consent forms, patient education materials, and medication directions. (3) *Communication *is how thoughts, messages or information are exchanged through speech, signals, writing or behavior. Communication involves inputs, decoding, encoding output, and feedback. Essential communication skills are reading with understanding, conveying ideas in writing, speaking so others can understand, listening actively, and observing critically.

In conclusion, the range of factors that are considered as key components of health literacy is extensive, and there is a wide variation between conceptual models. However, this diversity of views can to a large extent be reduced to two dimensions, notably the core qualities of health literacy (e.g., basic or functional, interactive, and critical health literacy), and its scope and area of application (e.g., as a patient in healthcare, as a consumer at the market, as a citizen in the political arena, or as a member of the audience in relation to the media).

#### Antecedents and consequences of health literacy

Apart from the dimensions of health literacy, the conceptual models summarized in Table 3 also give the main antecedents and consequences of health literacy outlined in the literature.

For the ***antecedents***, most authors refer to demographic, psychosocial, and cultural factors, as well as to more proximal factors such as general literacy, individual characteristics and prior experience with illness and the healthcare system. Among the demographic and social factors which impact on health literacy one notes socioeconomic status, occupation, employment, income, social support, culture and language [40], environmental and political forces [35], and media use [50]. In addition, peer and parental influences may impact on the health literacy of adolescents. In terms of personal characteristics, health literacy is predicted by age, race, gender and cultural background [50]; as well as by competences such as vision, hearing, verbal ability, memory and reasoning [40], physical abilities and social skills [50], and meta-cognitive skills associated with reading, comprehension, and numeracy [4,48,50]. The latter refers to the level of overall literacy, defined as the capacity to use printed and written information to function in society, achieve one's goals, and develop one's knowledge and potential. Finally, Nutbeam [36] points out that health literacy is also a result of health promotion actions such as education, social mobilization and advocacy.

In terms of the **consequences**, a number of researchers pointed out that health literacy leads to improved self-reported health status, lower healthcare costs, increased health knowledge, shorter hospitalization, and less frequent use of healthcare services [43,48,50,53]. According to Baker [49], these better health outcomes are caused by the acquisition of new knowledge, more positive attitudes, greater self-efficacy, and positive health behaviors associated with higher health literacy. Paashe-Orlow and Wolf [40] posit that health literacy influences three main factors which in turn have an impact on health outcomes: (1) navigation skills, self-efficacy and perceived barriers influence the access and utilization of healthcare; (2) knowledge, beliefs and participation in decision-making influence patient/provider interactions; and (3) motivation, problem-solving, self-efficacy, and knowledge and skills influence self care. The relationship of health literacy to health outcomes according to these authors must be conceived as a step function with a threshold effect, rather than in a simple linear fashion. People generally exist within a web of social relationships; and below a certain level of function, much of the day-to-day detail of chronic disease management often needs to be facilitated by others. While the interaction between health literacy and social support is likely to have complicated and subtle implications, the health impact of social effects has not been fully elucidated in the context of health literacy [54].

Nutbeam [36] distinguishes between individual and community or social benefits of health literacy. In terms of individual benefits, functional health literacy leads to an improved knowledge of risks and health services, and compliance with prescribed actions; interactive health literacy to an improved capacity to act independently, an improved motivation and more self-confidence; and critical health literacy to improved individual resilience to social and economic adversity. In terms of community and social benefits, functional health literacy increases the participation in population health programs; interactive health literacy enhances the capacity to influence social norms and interact with social groups; and critical health literacy improves community empowerment and enhances the capacity to act on social and economic determinants of health. Nutbeam's conceptual framework has been applied in case studies focusing on topics of diarrhea [55], self-management in diabetes [56] and health promoting schools [57].

Ratzan [58] links health literacy in the community to the concept of social capital, arguing that health literate people live longer and have stronger incentives to invest in developing their own and their children's knowledge and skills. Healthier populations tend to have higher labor market productivity contributing to, rather than withdrawing from, pension schemes. Similarly, healthier people use the health system less, and coupled with education and cognitive function, appropriately demand fewer health services.

### An integrated conceptual model of health literacy

**Figure 1:**
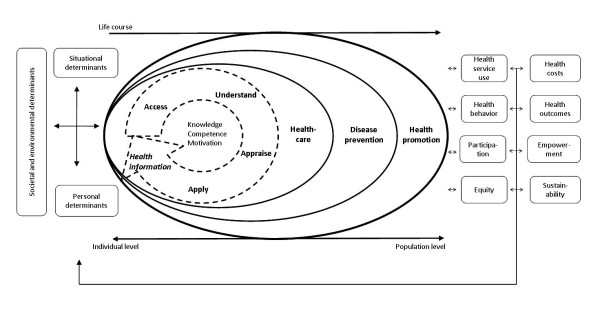
**Integrated model of health literacy--see separate file**.

Whereas a number of conceptual models of health literacy have been presented in the literature, none of these can be regarded as sufficiently comprehensive to line up with the evolving health literacy definitions and with the competencies they imply [59]. This is probably due to the fact that attempts to conceptualize health literacy have thus far failed to integrate the existing knowledge encompassing different perspectives on health literacy. Firstly, most of the existing conceptual models are not sufficiently grounded in theory in terms of the notions and concepts included. Secondly, very few models have integrated the components included in "medical" and "public health" literacy models. The only models which explicitly try to bridge the difference between both views are Nutbeam's [36] and Manganello's [50], whose dimension of functional literacy corresponds with the cognitive skills of medical health literacy. Thirdly, while acknowledging that health literacy entails different dimensions, the majority of the existing models are rather static and do not explicitly account for the fact that health literacy is also a process, which involves the consecutive steps of accessing, understanding, processing and communicating information. Fourthly, while most conceptual models identify the factors that influence health literacy and mention its impact on health service use, health costs and health outcomes, the pathways linking health literacy to its antecedents and consequences are not very clear. Researchers could link conceptual models of health literacy more explicitly to established health promotion theories and models [59]. Finally, very few conceptual models of health literacy have been empirically validated. To address these shortcomings, we propose an integrated model of health literacy which captures the main dimensions of the existing conceptual models reviewed above (Figure 1).

The model combines the qualities of a conceptual model outlining the main dimensions of health literacy (represented in the concentric oval shape in the middle of Figure 1), and of a logical model showing the proximal and distal factors which impact on health literacy, as well as the pathways linking health literacy to health outcomes.

The core of the model shows the competencies related to the process of accessing, understanding, appraising and applying health-related information. According to the 'all inclusive' definition this process requires four types of competencies: (1) *Access *refers to the ability to seek, find and obtain health information; (2) *Understand *refers to the ability to comprehend the health information that is accessed; (3) *Appraise *describes the ability to interpret, filter, judge and evaluate the health information that has been accessed; and (4) *Apply *refers to the ability to communicate and use the information to make a decision to maintain and improve health. Each of these competences represents a crucial dimension of health literacy, requires specific cognitive qualities and depends on the quality of the information provided [60]: obtaining and accessing health information depends on understanding, timing and trustworthiness; understanding the information depends on expectations, perceived utility, individualization of outcomes, and interpretation of causalities; processing and appraisal of the information depends on the complexity, jargon and partial understandings of the information; and effective communication depends on comprehension. The competences also incorporate the qualities of functional, interactive and critical health literacy as proposed by Nutbeam [36].

This process generates knowledge and skills which enable a person to navigate three domains of the health continuum: being ill or as a patient in the healthcare setting, as a person at risk of disease in the disease prevention system, and as a citizen in relation to the health promotion efforts in the community, the work place, the educational system, the political arena and the market place. Going through the steps of the health literacy process in each of these three domains equips people to take control over their health by applying their general literacy and numerical skills as well as their specific health literacy skills to acquire the necessary information, understanding this information, critically analyzing and appraising it, and acting independently to engage in actions overcoming personal, structural, social and economical barriers to health. As contextual demands change over time, and the capacity to navigate the health system depends on cognitive and psychosocial development as well as on previous and current experiences, the skills and competencies of health literacy develop during the life course and are linked to life long learning.

The frameworks associated with the three domains represent a progression from an individual towards a population perspective. As such, the model integrates the "medical" conceptualization of health literacy with the broader "public health" perspective. Placing greater emphasis on heath literacy outside of healthcare settings has the potential to impact on preventative health and reduce pressures on health systems.

The combination of the four dimensions referring to health information processing with the three levels of domains yields a matrix with 12 dimensions of health literacy as illustrated in Table 4.

**Table 4:** The matrix with four dimensions of health literacy applied to three health domains

	Access/obtain information relevant to health	Understand information relevant to health	Process/appraise information relevant to health	Apply/use information relevant to health
**Health care**	Ability to access information on medical or clinical issues	Ability to understand medical information and derive meaning	Ability to interpret and evaluate medical information	Ability to make informed decisions on medical issues

**Disease prevention**	Ability to access information on risk factors for health	Ability to understand information on risk factors and derive meaning	Ability to interpret and evaluate information on risk factors for health	Ability to make informed decisions on risk factors for health

**Health promotion**	Ability to update oneself on determinants of health in the social and physical environment	Ability to understand information on determinants of health in the social and physical environment and derive meaning	Ability to interpret and evaluate information on health determinants in the social and physical environment	Ability to make informed decisions on health determinants in the social and physical environment

Four dimensions of health literacy in the domain of *healthcare*, i.e., the ability to access information on medical or clinical issues, to understand medical information, to interpret and evaluate medical information, and to make informed decisions on medical issues and comply with medical advice.

Four dimensions of health literacy in the domain of *disease prevention*, notably the ability to access information on risk factors for health, to understand information on risk factors and derive meaning, to interpret and evaluate information on risk factors, and to make informed decisions on risk factors for health.

Four dimensions in the domain of *health promotion*, notably the ability to regularly update oneself on determinants of health in the social and physical environment, to comprehend information on determinants of health in the social and physical environment and derive meaning, to interpret and evaluate information on determinants, of health in the social and physical environment, and the ability to make informed decisions on health determinants in the social and physical environment.

Health literacy is in our understanding regarded an asset for improving people's empowerment within the domains of healthcare, disease prevention and health promotion.

In addition to the components of health literacy proper, the model in Figure 1 also shows the main antecedents and consequences of health literacy. Among the factors which impact on health literacy, a distinction is made between more distal factors, including societal and environmental determinants (e.g., demographic situation, culture, language, political forces, societal systems), and proximal factors, which are more concerned with personal determinants (e.g., age, gender, race, socioeconomic status, education, occupation, employment, income, literacy) and situational determinants (e.g. social support, family and peer influences, media use and physical environment). Health literacy is strongly associated with educational attainment [50], as well as with overall literacy [34,38,39]. Fundamental literacy affects a wide range of cognitive, behavioral, and societal skills and abilities. It should be distinguished from other specific literacy, such as science literacy (i.e., the ability to comprehend technical complexity, understanding of common technology, and an understanding that scientific uncertainty is to be expected), cultural literacy (i.e., recognizing and using collective beliefs, customs, world-views, and social identity relationships) and civic literacy (i.e., knowledge about sources of information and about agendas and how to interpret them, enabling citizens to engage in dialogue and decision-making). According to Mancuso [43], an individual must have certain skills and abilities to obtain competence in health literacy, and identifies six dimensions that are considered as necessary antecedents of health literacy, namely operational, interactive, autonomous, informational, contextual, and cultural competence.

Health literacy in turn influences health behavior and the use of health services, and thereby will also impact on health outcomes and on the health costs in society. At an individual level, ineffective communication due to poor health literacy will result in errors, poor quality, and risks to patient safety of the healthcare services [61]. At a population level, health literate persons are able to participate in the ongoing public and private dialogues about health, medicine, scientific knowledge and cultural beliefs. Thus, the benefits of health literacy impact the full range of life's activities--home, work, society and culture [34,38,39]. Advancing health literacy will progressively allow for greater autonomy and personal empowerment, and the process of health literacy can be seen as a part of an individual's development towards improved quality of life. In the population, it may also lead to more equity and sustainability of changes in public health. Consequently, low health literacy can be addressed by educating persons to become more resourceful (i.e., increasing their personal health literacy), and by making the task or situation less demanding, (i.e., improving the "readability of the system").

## Discussion

In this article we have we have presented a working definition of health literacy which represents the essence of the definitions of this concept as given in the literature. Furthermore a new conceptual model has been developed as a result of the review of existing health literacy concepts. While the literature indicates that health literacy refers to the competences of people to meet the complex demands of health in modern society [2,3,62] the exact nature of these competences is still debated. One perspective is that they refer to a series of individual cognitive skills and abilities applied in a medical context; the other perspective sees a broader range of competencies applied in the social realm. The first is referred to as "medical health literacy" [5], "patient health literacy" [14], or "clinical health literacy" [63]; the second as "public health literacy" [35]. Nutbeam [52] refers to the opposing medical and public health views on health literacy as respectively a "clinical risk", and a "personal asset" approach, and points out that they are rooted in the different traditions of clinical care, and adult learning and health promotion, respectively. As both perspectives are important and useful to enable a better understanding of health communication processes in clinical and community settings, any definition of health literacy needs to integrate both views. The proposed 'all inclusive' definition is adaptable and includes the public health perspective as well as the individual perspective.

While originating from the study of the reading and numerical skills that are necessary to function adequately in the healthcare environment, the concept of health literacy has expanded in meaning to include information-seeking, decision-making, problem-solving, critical thinking, and communication, along with a multitude of social, personal, and cognitive skills that are imperative to function in the health-system [49,52,59]. It has now diffused into the realm of culture, context, and language [49,52,59]. Although some authors have argued that health literacy is merely "new wine in old bottles", and is basically the repackaging of concepts central to the ideological theory and practice of health promotion [64], enhancing health literacy is increasingly recognized as a public health goal and a determinant of health. As new health literacy frameworks have emerged to clarify the deeper meaning of health literacy, its contribution to health, and the social, environmental, and cultural factors that influence health literacy skills in a variety of populations, there is a need for an integration of diverging definitions, conceptual frameworks and models of health literacy.

## Conclusion

The conceptual framework presented in this paper provides this integration in the form of a comprehensive model. Based on a systematic review of existing definitions and conceptualizations of health literacy, it combines the qualities of a conceptual model outlining the most comprehensive dimensions of health literacy, and of a logical model, showing the proximal and distal factors which impact on health literacy as well as the pathways linking health literacy to health outcomes. Specifically, the model identifies 12 dimensions of health literacy, referring to the competencies related to accessing, understanding, appraising and applying health information in the domains of healthcare, disease prevention and health promotion, respectively.

By integrating existing definitions and conceptualizations of health literacy into an encompassing model outlining the main dimensions of health literacy as well as its determinants and the pathways to health outcomes, this model has a heuristic value in its own right. More importantly, however, it can also support the practice of healthcare, disease prevention and health promotion by serving as a conceptual basis to develop health literacy enhancing interventions. Moreover, it can contribute to the empirical work on health literacy by serving as a basis for the development of measurement tools. As currently available tools to measure health literacy do not capture all aspects of the concept as discussed in the literature, there is a need to develop new tools to assess health literacy, reflecting health literacy definitions and accompanying conceptual models for public health. By following a concept validation approach, scales can be developed to assess the dimensions outlined in the conceptual model presented in this paper. This will not only produce a comprehensive measure of health literacy, reflecting the state of the art of the field and applicable for social research and in public health practice, but also serve to validate the conceptual model and thus contribute to the understanding of health literacy.

## Competing interests

The authors are members of the European Health Literacy (HLS-EU) consortium and claim to have no competing interests.

## Authors' contributions

Background: KS, SVDB, JF, GD. Methodology: KS, SVDB, JF. Results: KS, SVDB, HB, JF, GD, ZS and JP. Discussion: KS, SVDB, HB, JF, GD, ZS and JP. Conclusion: KS, SVDB, JF and GD. All authors read and approved the final manuscript.

## Pre-publication history

The pre-publication history for this paper can be accessed here:

http://www.biomedcentral.com/1471-2458/12/80/prepub
